# Fermented Corn–Soybean Meal Mixed Feed Modulates Intestinal Morphology, Barrier Functions and Cecal Microbiota in Laying Hens

**DOI:** 10.3390/ani11113059

**Published:** 2021-10-26

**Authors:** Yinglu Liu, Jia Feng, Yamin Wang, Jing Lv, Jinghe Li, Lijuan Guo, Yuna Min

**Affiliations:** College of Animal Science and Technology, Northwest A&F University, Yangling 712100, China; liuyinglu1996@163.com (Y.L.); fengjiacaas@163.com (J.F.); nwafuyummy@163.com (Y.W.); lvjing96@163.com (J.L.); z13389192603@126.com (J.L.); glj1430151801@163.com (L.G.)

**Keywords:** fermented feed, laying hen, cecal microbiota, gut health

## Abstract

**Simple Summary:**

Fermented feed has been of wide concern in livestock and poultry production because of its many advantages. In this study, the nutritional quality of the feed before and after fermentation was assessed, and four supplemental levels of fermented feed were used to replace unfermented feed to study the influence of fermented feed on the gut health of the laying hens during the laying peak period. The results suggest that fermented feed can improve the intestinal morphology and barrier functions of laying hens, possibly by altering the cecal microbiome.

**Abstract:**

This study aimed to evaluate the effects of fermented corn–soybean meal mixed feed on intestinal barrier function and cecal microbiota in laying hens. A total of 360 Jingfen No.6 laying hens (22 wk-old) were assigned to 4 dietary treatments, which were offered basal diets (without antibiotics) containing 0, 4, 6 and 8% of fermented mixed feed respectively. The results showed that the pH value and anti-nutritional factor concentrations in fermented mixed feed were lower than those in unfermented feed (*p* < 0.05). Moreover, fermentation in the feed significantly increased the crude protein content (*p* < 0.05). Supplementation with fermented feed significantly reduced the crypt depth and increased the villi height:crypt depth ratio of duodenum and jejunum (*p* < 0.05). Meanwhile, fermented feed increased the secretory immunoglobulin A content and MUC2 mRNA expression of jejunum (*p* < 0.05). These beneficial effects were exhibited at the addition level ≥6% and microbial composition of caeca in the control, and so 6% fermented feed groups were analyzed. The structure of the gut microbiota was remarkably altered by additions, characterized by increased abundances of some health-promoting bacteria, such as *Parasutterella*, *Butyricicoccus* and *Erysipelotrichaceae* (*p* < 0.05). In summary, fermented mixed feed modulated cecal flora, subsequently contributing to improvements in intestinal morphology and barrier functions in laying hens.

## 1. Introduction

To achieve resistance-free breeding, a growing number of studies has been conducted to evaluate non-antibiotic alternatives and their effects on the health and production performance of animals [1]. Fermented feed has attracted wide attention in livestock and poultry breeding because of their potential to improve the nutritional quality of feedstuffs by increasing nutrient bioavailability and reducing feed costs [2,3]. Previous research has demonstrated that fermentation may enhance the bioavailability of feed nutrients by the following processes: (1) increasing crude protein content [4], (2) decreasing fiber content, (3) enhancing the utilization of vitamins, (4) improving amino acid patterns and protein solubility [5] and (5) degrading anti-nutritional factors with enzymes, such as phytase, xylanase, cellulase, and glucanase enzymes [6]. Besides this, the probiotics and metabolites produced during the fermentation process could reduce the colonization of gut pathogenic microorganisms via competitive exclusion and the secretion of antibacterial substances (like bacteriocin), and thus exert beneficial effects on animals [7].

Gut integrity and function are essential factors in maintaining animal health and production performance. Intestinal morphology is an important criterion for evaluating intestinal health of animals. Villus height and crypt depth were related to nutrient digestion and poultry performance [8]. The composition and diversity of intestinal microorganisms also have a profound impact on the gut health of poultry. Colonization of harmful bacteria will disrupt the intestinal microbial balance, and then affect the host’s physiology, immunity, and nutritional metabolism [9]. Probiotic fermentation has been regarded as an effective method to enhance gut health [10] and it has been widely used in pig rations for several years [11]. Recently, there have been more studies on the utilization of fermented products in poultry industry, particularly focusing on gut health and production parameters of birds. It was reported that the use of fermented feed had a positive impact on the production performance and egg quality of 16 wk Babcock pullets [12]. Semjon et al. [13] observed that fermented wheat bran supplementation could improve broiler performance and meat quality. Fermented feed has been demonstrated to be beneficial to the maintenance of gut microbial ecosystems and intestinal morphology, possibly due to low pH, elevated numbers of probiotics, high short-chain fatty acid concentrations and reduced pathogens [14,15]. In addition, fermented feed could modulate gut microbiota by providing energy and nutrients to probiotics in the microbial community [16]. Therefore, fermented feed was speculated to exert beneficial effects on gut health by altering intestinal microbial composition and subsequently contributed to improvements in laying hen performance. However, the effects of fermented feed on intestinal community and gut health of laying hens remain unclear.

In China, fermented feed mainly refers to the fermented single feed material, such as soybean meal [17], rapeseed meal [15] and cottonseed meal [18]. However, there were few studies evaluating the effectiveness of corn–soybean meal mixed feed in laying hens. Based on the results of previous studies, we hypothesized that a certain concentration of fermented corn–soybean mixed feed could improve the gut health of laying hens. Therefore, the present study was carried out to assess the physicochemical characteristics of mixed feed following fermentation and then to investigate the effects of graded levels of fermented feed on gut morphology and mechanical and immunological barriers, as well as the cecal microbiota of laying hens.

## 2. Materials and Methods

### 2.1. Preparation of Fermented Mixed Feed

The probiotic purchased from Baide Biotechnology Co., Ltd. (Shandong, China), was a lyophilized powder containing *Bacillus* 2 × 10^9^ CFU/g, *Lactobacillus* 3 × 10^9^ CFU/g, *Saccharomyces cerevisiae* 5 × 10^8^ CFU/g. The probiotic powder was dissolved in 1 L sterile water at 37 °C, and stirred evenly to make a probiotic solution. The basal substrate (12 kg) included 60% corn, 20% soybean meal and 20% wheat bran, which was mixed and inoculated in probiotics with probiotic solution. The fermentation process according to the method of Shi et al. [19], with appropriate adjustments made to adapt to the actual situation of the experimental farm. Sterile water was added to the mixed substrate to reach a moisture content of 30%, and aerobic fermentation was carried out in a fermenter vessel at 37 °C for 24 h. After the first stage, a mixture of aerobic fermented mixture was transferred to a plastic drum equipped with a gas-pressure opening valve for 37 °C anaerobic fermentation, then fermented under anaerobic conditions at 37 °C for 5 days (the second stage of fermentation). The same proportion of 37 °C sterile water was added to the unfermented mixture and sealed for 5 days as control. Fermented feed was produced every week, and the feed intake of laying hens was measured once a week to adjust the amount of fermented feed.

### 2.2. Chemical Analysis of Fermented Mixed Feed

Fermented and unfermented feed were collected and dried at 65 °C for 48 h. Feed samples (*n* = 3) were tested to determine their crude fiber (CF; AOAC #978.10), crude protein (CP; AOAC #984.13) and ether extract (EE; AOAC #2003.05) contents according to AOAC International guidelines [20]. Phytic acid levels were determined as in a previous study [21]. Trypsin inhibitor and β-glucan in fermented and unfermented mixture were tested using a commercial kit (Jianglai Bio Company, Shanghai, China). To determine pH, 5 g of fermented and unfermented mixed feed were dissolved in 50 mL distilled water. After centrifuging at 4000× *g* for 5 min, the supernatant pH was tested using a probe style-pH meter (H170 Hach pH meter, Hach, Loveland, CO, USA). Protein extraction and SDS-polyacrylamide gel electrophoresis (PAGE) of the feed samples were performed according to the method described in reference [22].

### 2.3. Birds, Housing and Dietary Treatments

The trial was carried out in the non-antibiotic breeding demonstration plant of Chunmanyuan Farm (Tongchuan, Shaanxi, China). A total of 360 Jingfen No.6 layers aged 22 weeks were randomly assigned to four numerically equal groups, with 6 replicates per treatment and 15 birds per replicate. The layers had a similar body weight and good health. The feeding period lasted 10 weeks, commencing when the layers were 25 weeks of age and ending when they were 35 weeks of age, with an addition of 3 weeks for feed adaptation. During the experiment, three laying hens were kept in each cage and had ad libitum access to feed and water. Proper indoor temperature (15~22 °C) and humidity (30%~50%) were maintained.

According to the NRC (1994) layer feeding standard, the corn–soybean meal basal diet (antibiotic-free) was designed based on the actual situation of layer feeding in the experimental chicken farm. Unfermented corn, soybean meal, and wheat bran were replaced in the basic diet with increasing levels of fermented mixed feed (0, 4%, 6% and 8%; F0, F4, F6 and F8, respectively). The ingredients were mixed in a mixer for 15 min. Feed composition and nutrient content of the experimental diets are shown in Table 1. 

### 2.4. Sample Collection

At 35 wk, 24 chickens (one with an average body weight from each replicate pen) were selected to collect samples. All birds were killed by cervical dislocation and small intestinal (duodenum, jejunum and ileum) mucosa was scraped off at the forepart of individual small intestinal segments with a glass microscope slide on ice and frozen and stored immediately at −80 °C liquid nitrogen tanks for further analysis. About 3 cm of the mid-portion of the small intestines were excised carefully and washed in saline solution before storing in formalin (10%). The cecal contents were gathered using sterile spatulas into sterile plastic tubes, immediately refrigerated (maximum 2 h) and stored at −80 °C refrigeration until the DNA extraction. 

### 2.5. Morphological Investigations

Three-centimeter lengths from the medial portions of duodenum, jejunum and ileum from chickens were fixed with 10% formalin for 24 h. Tissues were later embedded in paraffin wax blocks, mounted onto glass slides, and then stained with Haematoxylin & Eosin (H&E). The fixed segments were sectioned and observed under the light microscope, and the villus height VD, crypt depth CD and the ratio of VD and CD evaluated.

### 2.6. Enzyme-Linked Immunosorbent Assay (ELISA) and Real-Time Polymerase Chain Reaction (PCR)

Secretory immunoglobulin A (sIgA) content of mucous membrane samples of the duodenum, jejunum, and ileum were measured by ELISA kits (Cloud-Clone Crop Biological Technology Co., Ltd., Wuhan, China) according to the kit instructions. Total RNA was extracted from the snap-frozen jejunal tissue samples with a RNeasy mini kit (Qiagen, Germantown, MD, USA), following the instructions. Complementary DNA (cDNA) was synthesized from 1 μg of total RNA using MMLV reverse transcriptase (TaKaRa, Dalian, China). Then, the transcriptional changes were identified by quantitative PCR using Premix Ex TaqTM with SYBR Green (TaKaRa, Dalian, China) and the Bio-Rad CFX 96™ Real-Time Detection System (Bio-Rad Laboratories, Richmond, CA, USA). The thermocycle protocol lasted for 30 s at 94 °C, followed by 40 cycles of 5 s denaturation at 94 °C, 34 s annealing/extension at 60 °C, and then a final melting curve analysis to monitor the purity of the PCR product. The 2−ΔΔCt method was used to estimate mRNA abundance (Livak et al., 2002). Relative gene expression levels were normalized using β-actin as an internal control. The primers were synthesized by the Xi’an Qingke Biological Company, and the sequences are shown in Table 2.

### 2.7. 16S rRNA Gene Sequencing

Total genomic DNA was extracted from samples using the CTAB/SDS method and then stored at −80 °C until sequencing analysis. The V3 + V4 fragments of the 16S rRNA gene were amplified using the Phusion^®^ High-Fidelity PCR Master Mix (New England Biolabs, Cambridge, MA, USA) and primers F341 and R806. The 30 μL reaction system was used in PCR reactions with 15 μL Mix, 0.2 M of forward and reverse primers, and about 10 ng template DNA. Thermal cycling consisted of initial denaturation at 98 °C for 1 min, followed by 30 cycles of denaturation at 98 °C for 10 s, annealing at 50 °C for 30 s, elongation at 72 °C for 30 s and a final elongation at 72 °C for 5 min. The amplicons were examined using 2% agarose gel electrophoresis, and then the GeneJET Gel Extraction Kit was used (Thermo Fisher Scientific, Waltham, MA, USA) to purify the excised target fragments. Finally, 16S rRNA gene sequencing was performed using the Illumina NovaSeq 6000 PE250 (Illumina, Santa Clara, CA, USA) with the MiSeq Reagent Kit from Novogene Bioinformatics Technology Co., Ltd., Beijing, China.

### 2.8. Statistical Analysis

Alpha diversity (Shannon and Simpson indices) and abundance (Chao1 and ACE indices) were analyzed withQiime v.1.7.1. (http://qiime.org/index.html). Data regarding alpha diversity indices, intestinal morphology, total sIgA concentration and relative mRNA expression levels among all treatments in this study were analyzed by one-way ANOVA with SPSS 22.0. Differences among groups means were determined by Duncan’s multiple comparison test. The significance of differentiations in microbial structure among groups was assessed by ANOSIM using R package “vegan”. *t*-test was used to compare the chemical composition of feed and cecal microflora at the phylum and genus level. The correlations between the cecal microbial composition and gut health were assessed by Spearman’s correlation analysis using GraphPad Prism version 8.00. *p* < 0.05 was considered statistically significant and 0.05 < *p* ≤ 0.10 a trend.

## 3. Results

### 3.1. Chemical Composition of Fermented Mixed Feed 

**Figure 1:**
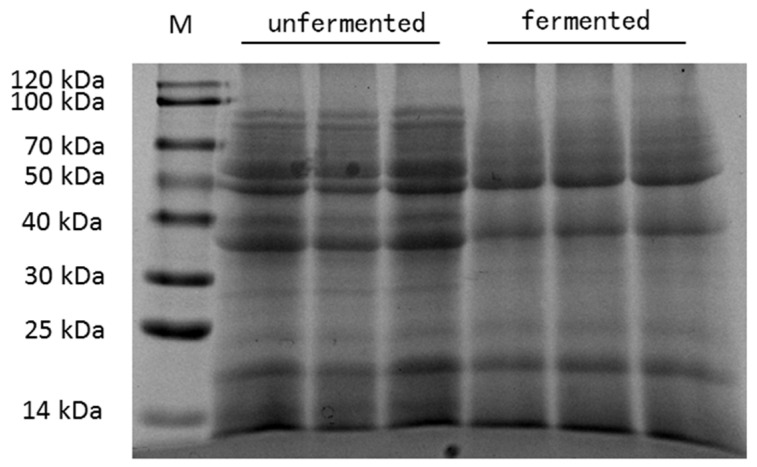
SDS-PAGE map of unfermented mixed feed and fermented mixed feed. M = protein marker.

The chemical composition of unfermented mixed feed and fermented mixed feed are summarized in Table 3. The pH value and phytic acid, trypsin inhibitor and β-glucan concentrations in fermented mixed feed were lower than in unfermented mixed feed. Moreover, the fermentation of feed significantly increased the crude protein content. Furthermore, there were no significant differences of the total ether extract and crude fiber among the two different kinds of feed. As shown in Figure 1, unfermented mixed feed contained greater amounts of large- (>60 kDa) and medium-size (20–60 kDa) peptide than fermented mixed feed.

### 3.2. Intestinal Morphology

The measurement of intestinal morphology is a common way to judge the integrity and function of the intestinal barrier. The results on villi height (VH), crypt depth (CD) and the villi height:crypt depth ratio (VH/CD) are displayed in Table 4. In the duodenum, three levels of fermented mixed feed significantly decreased the CD and increased the VH/CD ratio. In the jejunum, dietary supplementation with fermented mixed feed significantly increased the VH and VH/CD ratio compared to the control group. However, the 4% fermented mixed feed group significantly increased CD compared with all other treatments. In the ileum, no significant effect of dietary fermented mixture supplementation was observed on the VH, CD and VH/CD ratio.

### 3.3. Total sIgA Concentration and Physical Barrier mRNA Abundance in the Intestinal Mucosa

**Figure 2:**
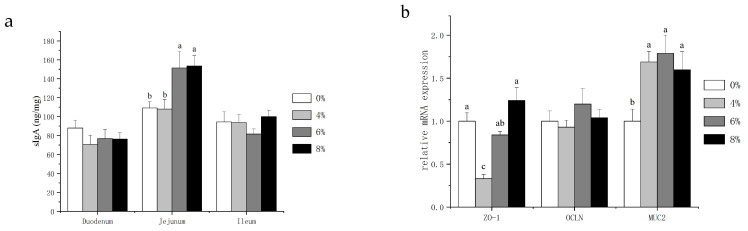
Effects of fermented mixture supplementation on the intestinal sIgA content (**a**) and the physical barrier in the jejunum (**b**) of laying hens. Data are expressed as mean ± SEM of 6 replicates per treatment; ^a–c^ There are statistically significant differences in the mean values per line for different superscripts (*p* < 0.05).

Secretory IgA (sIgA) acts as the first immune defense for intestinal epithelium and maintains the homeostasis of the gut. Therefore, we assessed the intestinal immune function by measuring the intestinal sIgA contents. As shown in Figure 2a, no statistical differences in sIgA content were observed in the duodenum and ileum. However, the jejunal sIgA content was significantly higher in the 6% and 8% fermented mixed feed group compared to the other treatments. In the presence of intact epithelial cell layers, intercellular paracellular pathways must be closed. This function is effectuated through physical barriers—especially by tight junctions [23]. In addition, mucin 2 (MUC2) is the most abundant mucin, which creates the first defense line against invading microorganisms [24]. Thus, we focused on the mRNA expression levels of zonula occludens 1 (ZO-1), occludin (OCLN) and MUC2 in jejunal mucosa to further explore the physical barriers of laying hens. Figure 2b showed that fermented mixed feed at three levels all significantly increased MUC2 gene expression compared with the control group, whereas 4% fermented mixed feed significantly decreased the mRNA expression of ZO-1.

### 3.4. Cecal Microbial Diversity and Community

**Figure 3:**
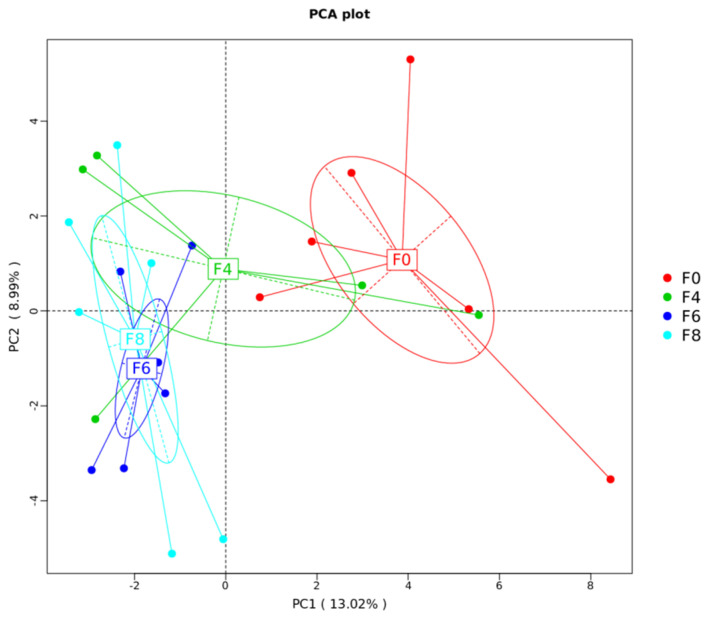
Comparison of the compositions of the cecal microbiota by principal component analysis (PCA). F0, basal diet; F4, 4% fermented mixed feed; F6, 6% fermented mixed feed; F8, 8% fermented mixed feed.

As the microbial community plays an important role in the intestinal health and barrier function of laying hens, the composition of the cecal microbial community was analyzed using the 16S rRNA gene amplicon sequence. After filtering, an average of 60,788 ± 6481 reads were obtained for each sample. In the 16S amplicon data analysis, the Shannon and Simpson indices were used to assess the community diversity, and the ACE and Chao1 indices reflected the community richness. Therefore, alpha diversity was measured using these four indices, and there were no significant differences in cecal feces microbiome taxon abundance and diversity (*p* > 0.05; Table 5) among the groups, indicating that fermented mixed feed did not change the alpha diversity of the microbiota of the chicken cecum. Beta diversity was illustrated via principal component analysis (PCA) in Figure 3, showing fermented mixed feed treatment significantly affected cecal microbiota composition. The composition of the microbiota was similar between the 6% and 8% fermented mixed feed group. ANOSIM results, shown in Table 6, indicated that the variations of the inter-group microbiota composition of the 6% and 8% group were considered significant (*p* < 0.05) to the control group and were larger than those of the inner-group (R > 0.05). Hence, to illuminate how fermented mixed feed served to improve the gut health of laying hens, the 6% group was selected as the representative group among the 3 treated groups to identify the roles of fermented mixed feed in regulating the cecal microbiota.

**Figure 4:**
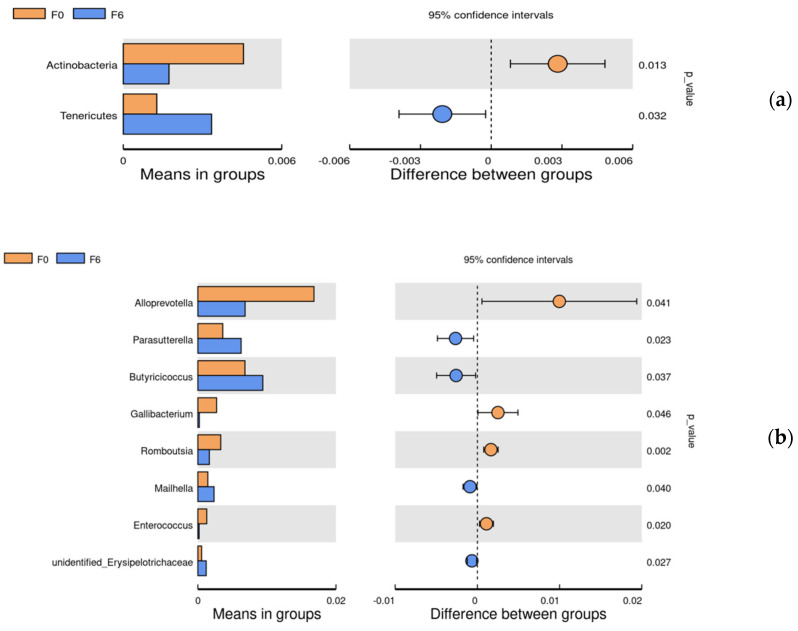
The phylum (**a**) and genera (**b**) differentially abundant between F0 and F6 in the cecal by *t*-test analysis. F0, basal diet; F6, 6% fermented mixed feed.

Since pairwise comparisons (0 and 6%) and the data were in accordance with normal distribution, *t*-test analysis was used to evaluate the differential bacteria (relative abundance > 0.1%) on the phylum and genus (Figure 4). Compared with the basal diet, at the phylum level, supplementation with 6% fermented mixed feed significantly improved the *Tenericutes* abundance but reduced the abundance of *Actinobacteria*; at the genus levels, the abundance of *Parasutterella*, *Butyricicoccus*, *unidentified_Erysipelotrichaceae* and *Mailhella* were found to be significantly increased and *Alloprevotella*, *Gallibacterium*, *Romboutsia* (*p* < 0.05) and *Enterococcus* were significantly decreased in the 6% fermentation group when compared with the 0% control group (*p* < 0.05).

### 3.5. Correlations between Microbiota and Gut Health

**Figure 5:**
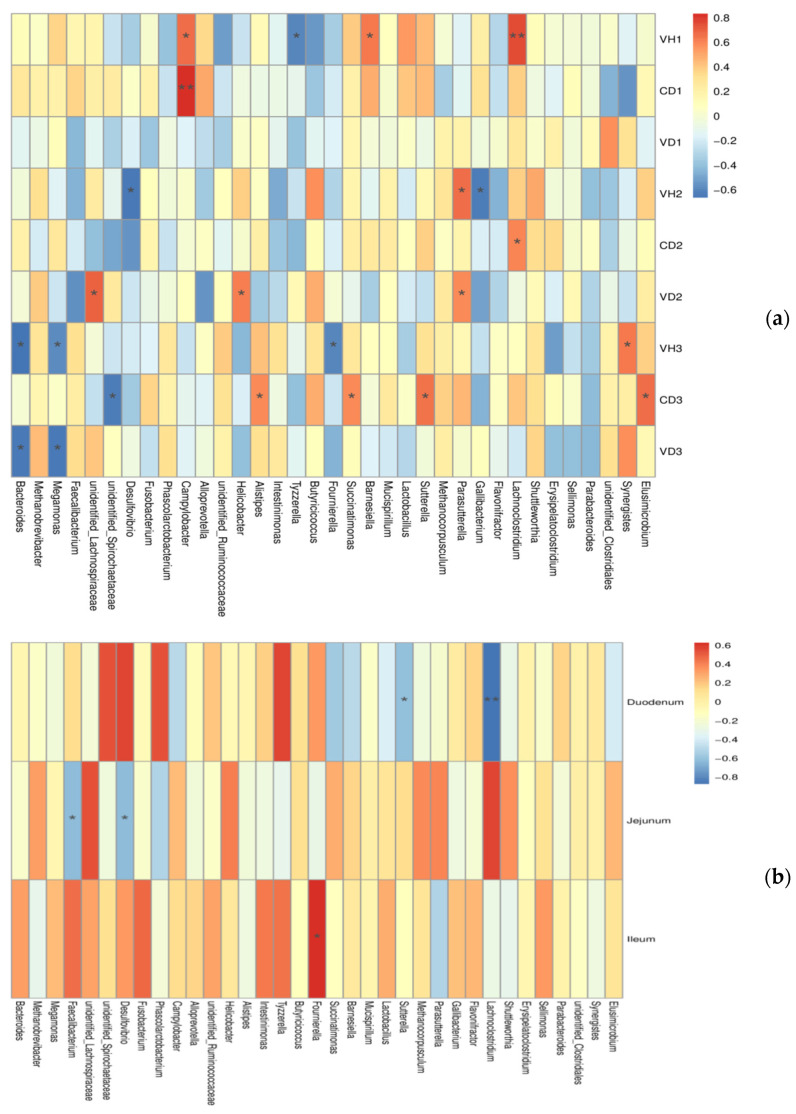
Heatmap of spearman’s correlation between the microbiota from chicken cecum and gut health. (**a**) cecal microbiota and intestinal morphology; (**b**) cecal microbiota and sIgA contents of intestine. * *p* < 0.05, ** *p* < 0.01. Red and blue cells indicate positive and negative correlations, respectively. Color intensity is in proportion to magnitude.

In order to explore the specific bacteria related to gut health, the Spearman correlation coefficient (Figure 5) was used to analyze the correlation between the abundance of the cecal microbiota and the intestinal barrier. This analysis can identify species that are significantly correlated with certain environmental factors. The heatmap revealed significant positive correlations between intestinal morphology (higher villi height VH, shorter crypt depth CD and lager villi height:crypt depth VH/CD indicate superior development) and *unidentified_Lachnospiraceae*, *unidentified_Spirochaetaceae, Barnesiella Helicobacter*, *Parasutterella* and *Synergistes*. In contrast, the intestinal morphology was negatively correlated with *Bacteroides*, *Megamonas*, *Desulfovibrio*, *Alistipes*, *Tyzzerella*, *Fournierella Succinatimonas*, *Gallibacterium* and *Elusimicrobium* significantly. In addition, the abundance of genus *Faecalibacterium* and *Desulfovibrio* were negatively correlated with the jejunal sIgA contents, and the abundance of genus *Sutterella* and *Lachnoclostridium* were negatively correlated with the duodenal sIgA content. Moreover, *Fournierella* was positively correlated with ileal sIgA contents. 

## 4. Discussion

The corn–soybean meal diet is the most widely applied in poultry production in China. However, ordinary corn–soybean meal diets contain some anti-nutritional factors, for instance, phytic acid, soybean antigenic protein and soy oligosaccharides, which may compromise the nutrient bioavailability and exhibit negative effects on animal health [25]. Solid-state fermentation was proposed to improve the nutritional properties of coarse plant materials and improve their application in animal feeds [26].

Among a variety of parameters, pH is an important reference to estimate the quality of fermented feed, because lower pH may favor the digestion of intestinal nutrients and inhibit the growth of pathogenic microorganisms in feed [27]. Our results showed that the pH of mixed feed following fermentation decreased from 6.23 to 4.49, indicating that the fermented feed has reached a low pH requirement. In addition, compared to the control, the fermented feed contained higher concentrations of crude protein, which was similar to previous research [28]. The increase in CP concentration might be related to the synthesis of microbial protein or the loss of dry matter during fermentation. Furthermore, the decreased contents of phytic acid, trypsin inhibitor and β-glucan in mixed feed were observed after fermentation, which have been considered as the major anti-nutritional factors in the current feed industry negatively affecting nutrient digestion and absorption of laying hens. A decreased amount of phytic acid in fermented feed may be due to the production of phytase by microorganisms [29]. The reduction of β-glucan could be a possible reason for the soluble fraction of the polymer [30]. Probiotics in fermented feed could decompose proteins, including trypsin inhibitors, through the secretion of proteases, which may have a beneficial impact on intestinal health [31]. In addition, SDS-PAGE analysis showed that large-sized protein contents in fermented mixed feed were reduced, consistent with the study of Shi et al. [19]. This indicated that large-sized proteins can be degraded into small-sized proteins or peptides during fermentation, presumptively assisting intestinal protein digestion in laying hens [32]. 

This study found that fermented mixed feed could partially improve the intestinal morphology of laying hens. This was consistent with the past study of Missotten et al. [33], who noticed higher villi height, lower crypt depth and increased villus crypt ratios in broilers fed fermented moist feed. Similar results were also shown by Li et al. [34] in the duodenum and jejunum of broilers fed 10% fermented soybean meal. Gut morphology parameters, including villus height VH, crypt depth CD, and villus crypt VH/CD ratios, are regarded as gold standards for assessing intestinal health status [8]. The increase of villi height suggested a greater area addressed to the absorption of available nutrients [35], which was conducive to enhanced intestinal function. On the contrary, shortening of villi and deepening of crypts could cause malabsorption of nutrients and consequently compromise the production performance of laying hens [36]. The positive impact of fermented feed on gut morphology is likely attributed to its regulatory roles in gut microbiota balance and microbial metabolites, which can promote enterocyte differentiation and proliferation [37]. It might be also related to the reduction of anti-nutrient factors in the fermented feed, as supported by the negative correlation between trypsin inhibitor in soybean meal and villi height [38]. Surprisingly, fermented mixed feed addition at 4% has opposite effects on jejunum morphology (i.e., the increased villi height and crypt depth) and the exact reasons for this phenomenon need further exploration.

The gut barrier can prevent the intestinal tract from the colonization of pathogens, and sIgA is the critical component of the immune barrier, limiting epithelial contact with pathogens and other antigens [39]. In this study, we observed a significant improvement in jejunum sIgA concentration with the addition of 6% and 8% fermented feed, indicating positive effects of fermented feed on the barrier function of gut mucosa to reduce the adverse effects on gut health. This was in combination with the improvements in jejunum morphology, suggesting that the fermented feed played a role in the intestinal mucosa health in the jejunum of laying hens. Jejunum, the longest part of the small intestine with the longest retention time of nutrients [40], is considered the best segment for exerting favorable effects of fermented feed on gut health. Therefore, the jejunum was chosen for further study. OCLN and ZO are unique proteins that form the extracellular barrier of the gut [41]. This barrier is famous for tight junctions, which make up a wall against invading pathogens in the intestine [42]. MUC2, the main mucin of the intestinal mucosa, is involved in providing nutrients and attachment sites for host bacteria, and it can contribute to selecting species-specific gut microbiota [43]. Little information is available on physical barrier gene expression in birds fed with fermented feed. This study indicated that fermented feed can apparently increase the gene expression of MUC2, suggesting that fermented feed may prevent intestinal epithelial cells from pathogen invasion by modulating MUC2 expression in the jejunum. Fermentation products, including probiotics [44] and organic acids [45], have been proven to significantly improve the expression of poultry gut barrier-related genes, but the mechanism of their action is complex and needs to be studied in depth. However, this study showed no significant effects on the expression of OCLN. In particular, the addition of 4% fermented mixed feed significantly downregulated ZO-1 gene expression, in accordance with the observations that 4% fermented feed significantly increased crypt depth. It is necessary to research an in vivo pathogenic attack model as a further study to confirm the influences of fermented feed on gut barrier functions.

To further explore the underlying mechanism of its modulation on gut health, we have used Illumina MiSeq sequencing to analyze the cecal microbiota. The present study revealed no significant effect on the α-diversity of the cecal microbiota, whereas the β-diversity analysis showed significant clustering between the 6% and 8% groups and controls, indicating that the cecal microorganism community profiles in the 6% and 8% fermented groups could be altered following fermented feed addition. This was in line with the results on the intestinal morphology and the barrier-related gene expression, indicating that the 6% and 8% fermented feed could significantly improve intestinal health with no remarkable difference between the two groups. It was possible that fermented mixed feed at levels above a certain threshold (which was 6% in this study) could result in changes of the gut microflora and subsequently improve intestinal morphology and barrier functions. Therefore, control and 6% groups were selected for further analysis. At the genus level, *Parasutterella*, *Butyricicoccus*, *unidentified_Erysipelotrichaceae* and *Mailhella* were identified as the main microbes with increased abundances in response to the addition of fermented feed. The increased proportion of *Parasutterella* has been reported to be beneficial to intestinal mucosal homeostasis [46]. Similarly, our results also confirmed a positive correlation between *Parasutterella* abundance and villus height VH and VH/CD ratio. *Butyricicoccus*, as butyrate producers, are assumed to improve growth performance, inhibit the proliferation of pathogens and relieve intestinal inflammation in broilers [47]. *Erysipelotrichaceae* may be associated with the degradation of feed ingredients and the production of short-chain fatty acids [48]. On the other hand, the decreased abundances of *Alloprevotella*, *Gallibacterium*, *Romboutsia* and *Enterococcus* were observed in 6% fermented feed group compared to the control. *Gallibacterium* has been recognized as a main cause of peritonitis and salpingitis in laying hens [49], which leads to decreased productive performance. *Alloprevotella* is known as an opportunistic pathogen; however, previous studies have noted that the increased abundance of *Alloprevotella* genera was linked to better intestinal health [50]. Besides this, the genus *Romboutsia* is a valuable intestinal biomarker maintaining host health, and *Enterococcus* with natural antimicrobial probiotic properties could prevent diarrhea in animal production [51]. However, their roles in gut health and functions of birds need to be explored. *Gallibacterium* is directly associated with poultry intestinal disease, which was also confirmed in this study by a negative relationship between abundance and villus height [52]. Therefore, the ameliorated gut morphology and enhanced epithelial barrier functions might be mainly attributed to the increased abundances of some health-promoting bacteria in the fermented feed supplementation group. Further investigations based on metabolomics should be done to detect changes in metabolites caused by fermentation and their effects on gut health in a future study.

## 5. Conclusions

In conclusion, fermented mixed feed could improve the morphology and barrier functions of the intestine, and alter the cecal microflora. This study demonstrated that fermented mixed feed could be used as a novel feed ingredient for laying hens, and that their favorable effects could be exhibited at addition levels of ≥6%. The specific mechanism of fermented feed-changing of cecal microflora of laying hens needs further study.

## Figures and Tables

**Table 1:** Composition and nutrient levels of experimental diets (air-dry basis, %).

Items	Group			
	0	4%	6%	8%
Ingredients				
Corn	56.00	56.00	56.00	56.00
Soybean meal	20.60	20.60	20.60	20.60
Wheat bran	2.40	2.40	2.40	2.40
Fermented feed	0.00	4.00	6.00	8.00
Unfermented feed	8.00	4.00	2.00	0.00
Stone powder	7.50	7.50	7.50	7.50
Soybean oil	0.50	0.50	0.50	0.50
Premix ^1^	5.00	5.00	5.00	5.00
Nutrient levels ^2^				
ME(MJ/kg)	12.09	12.09	12.09	12.09
Crude protein	15.99	16.00	16.01	16.01
Calcium	3.60	3.60	3.60	3.60
Total phosphorus	0.42	0.42	0.42	0.42
Lys	0.94	0.94	0.94	0.94
Met	0.44	0.44	0.44	0.44
Thr	0.70	0.70	0.70	0.70

^1^ The premix composed of: Vitamin A 10,000 IU, Vitamin D 31, 800 IU, Vitamin E 10 IU, Vitamin K 10 mg, Vitamin B 125 ug, Vitamin B1 l mg, Vitamin B24.5 mg, calcium pantothenate 50 mg, niacin 24.5 mg, pyridoxine 5 mg, biotin 1 mg, folic acid 1 mg, choline 500 mg, iodine 0.4 mg, ferrum 80 mg, copper 8 mg, selenium 0.3 mg. ^2^ Crude protein of the 0 group was measured, and the rest was calculated.

**Table 2:** Gene name, primer sequences.

Gene	Primers Sequence (5′–3′)
β-actin	F: ACACCCACACCCCTGTGATGAA
	R: TGCTGCTGACACCTTCACCATTC
ZO-1	F: TATAGAAGATCGTGCCGCCTCC
	R: GAGGTCTGCCATCGTAGCTC
Occludin	F: ACAGCCCTCAATACCAGGATGTG
	R: ACCATGCGCTTGATGTGGAA
MUC2	F: TTCATGATGCCTGCTCTTGTG
	R: CCTGAGCCTTGGTACATTCTTGT

F = forward primer; R = reverse primer.

**Table 3:** Chemical composition of unfermented mixture and fermented mixture.

	Unfermented Mixed Feed	Fermented Mixed Feed	SEM	*p*-Value
pH	6.23 ^a^	4.49 ^b^	0.40	<0.01
Crude protein, %	15.90 ^b^	16.18 ^a^	0.07	0.016
Crude fiber, %	3.33	3.20	2.80	0.680
Ether extract, %	0.57	1.09	0.15	0.060
Phytic acid, %	0.65 ^a^	0.37 ^b^	0.07	0.025
Trypsin inhibitor, μg/g	359.29 ^a^	216.39 ^b^	32.04	<0.01
β-glucan, μg/g	1588.89 ^a^	1204.32 ^b^	86.35	<0.01

Composition of fermented mixture: corn 60%, soybean meal 20%, wheat bran 20%;. ^a,b^ There are statistically significant differences in the mean values per line for different superscripts (*p* < 0.05).

**Table 4:** Effect of fermented mixed feed on intestinal morphology in laying hens.

	Fermented Mixed Feed				SEM	*p*-Value
	0%	4%	6%	8%		
Duodenum						
VH ^1^, μm	1767.04	1731.68	1774.26	1753.69	21.83	0.889
CD ^2^, μm	294.56 ^a^	256.96 ^b^	254.26 ^b^	221.31 ^c^	4.24	<0.01
VH/CD ^3^, μm/μm	6.18 ^bc^	6.86 ^b^	7.16 ^b^	8.02 ^a^	0.13	<0.01
Jejunum						
VH, μm	922.89 ^c^	1315.79 ^a^	1079.89 ^b^	1121.96 ^b^	19.41	<0.01
CD, μm	177.47 ^b^	222.91 ^a^	180.14 ^b^	169.85 ^b^	3.94	<0.01
VH/CD, μm/μm	5.28 ^b^	6.23 ^a^	6.28 ^a^	6.88 ^a^	0.12	<0.01
Ileum						
VH, μm	882.98	936.10	968.38	961.42	15.84	0.208
CD, μm	136.40	145.14	145.96	138.04	1.97	0.201
VH/CD, μm/μm	6.57	6.64	6.63	7.13	0.11	0.241

^1^ VH: villi height. ^2^ CD: crypt depth. ^3^ VH/CD: villi height: crypt depth ratio. ^a–c^ There are statistically significant differences in the mean values per line for different superscripts (*p* < 0.05).

**Table 5:** Effect of fermented mixed feed on α-diversity of cecal microflora in layers.

	Fermented Mixed Feed				SEM	*p*-Value
	0%	4%	6%	8%		
Chao1	693.32	682.26	712.44	731.14	10.61	0.398
Ace	702.49	694.70	723.97	740.77	10.52	0.419
Shannon	6.42	5.90	6.41	6.68	0.12	0.137
Simpson	0.97	0.94	0.96	0.97	0.01	0.287

**Table 6:** Comparison of similarities in microbiota composition between the three treatments by ANOSIM analysis.

Treatment	R-Value	*p*-Value
F0–F4	0.07407	0.4
F0–F8	0.6	0.016
F4–F8	0.4769	0.025
F0–F6	0.3457	0.045
F4–F6	0.2099	0.214
F6–F8	−0.02667	0.563

## Data Availability

Informed consent was obtained from all subjects involved in the study. The datasets analyzed in the present study are available from the corresponding author on reasonable request.

## References

[1] Khalique A., Zeng D., Shoaib M., Wang H., Qing X., Rajput D.S., Pan K., Ni X. (2020). Probiotics mitigating subclinical necrotic enteritis (SNE) as potential alternatives to antibiotics in poultry. AMB Express.

[2] Wang J., Han Y., Zhao J.-z., Zhou Z.-j., Fan H. (2017). Consuming fermented distillers’ dried grains with solubles (DDGS) feed reveals a shift in the faecal microbiota of growing and fattening pigs using 454 pyrosequencing. J. Integr. Agric..

[3] Plumed-Ferrer C., von Wright A. (2009). Fermented pig liquid feed: Nutritional, safety and regulatory aspects. J. Appl. Microbiol..

[4] Wang C., Shi C., Su W., Jin M., Xu B., Hao L., Zhang Y., Lu Z., Wang F., Wang Y. (2020). Dynamics of the Physicochemical Characteristics, Microbiota, and Metabolic Functions of Soybean Meal and Corn Mixed Substrates during Two-Stage Solid-State Fermentation. mSystems.

[5] Wang T.Y., Wu Y.H., Jiang C.Y., Liu Y. (2010). Solid state fermented potato pulp can be used as poultry feed. Br. Poult. Sci..

[6] Taheri H.R., Moravej H., Tabandeh F., Zaghari M., Shivazad M. (2009). Screening of lactic acid bacteria toward their selection as a source of chicken probiotic. Poult. Sci..

[7] Jin W., Zhang Z., Zhu K., Xue Y., Xie F., Mao S. (2019). Comprehensive Understanding of the Bacterial Populations and Metabolites Profile of Fermented Feed by 16S rRNA Gene Sequencing and Liquid Chromatography-Mass Spectrometry. Metabolites.

[8] Ducatelle R., Goossens E., De Meyer F., Eeckhaut V., Antonissen G., Haesebrouck F., Van Immerseel F. (2018). Biomarkers for monitoring intestinal health in poultry: Present status and future perspectives. Vet. Res..

[9] Hiippala K., Jouhten H., Ronkainen A., Hartikainen A., Kainulainen V., Jalanka J., Satokari R. (2018). The Potential of Gut Commensals in Reinforcing Intestinal Barrier Function and Alleviating Inflammation. Nutrients.

[10] Sugiharto S., Ranjitkar S. (2019). Recent advances in fermented feeds towards improved broiler chicken performance, gastrointestinal tract microecology and immune responses: A review. Anim. Nutr..

[11] Canibe N., Jensen B.B. (2012). Fermented liquid feed-Microbial and nutritional aspects and impact on enteric diseases in pigs. Anim. Feed Sci. Technol..

[12] Engberg R.M., Hammershoj M., Johansen N.F., Abousekken M.S., Steenfeldt S., Jensen B.B. (2009). Fermented feed for laying hens: Effects on egg production, egg quality, plumage condition and composition and activity of the intestinal microflora. Br. Poult Sci..

[13] Semjon B., Bartkovsky M., Marcincakova D., Klempova T., Bujnak L., Hudak M., Jaduttova I., Certik M., Marcincak S. (2020). Effect of Solid-State Fermented Wheat Bran Supplemented with Agrimony Extract on Growth Performance, Fatty Acid Profile, and Meat Quality of Broiler Chickens. Animals.

[14] Xie Y., Liu J., Wang H., Luo J., Chen T., Xi Q., Zhang Y., Sun J. (2020). Effects of fermented feeds and ginseng polysaccharides on the intestinal morphology and microbiota composition of Xuefeng black-bone chicken. PLoS ONE.

[15] Chiang G., Lu W.Q., Piao X.S., Hu J.K., Gong L.M., Thacker P.A. (2009). Effects of Feeding Solid-state Fermented Rapeseed Meal on Performance, Nutrient Digestibility, Intestinal Ecology and Intestinal Morphology of Broiler Chickens. Asian Australas. J. Anim. Sci..

[16] Wang C., Shi C., Zhang Y., Song D., Lu Z., Wang Y. (2018). Microbiota in fermented feed and swine gut. Appl. Microbiol. Biotechnol..

[17] Mathivanan R., Selvaraj P., Nanjappan K. (2006). Feeding of Fermented Soybean Meal on Broiler Performance. Int. J. Poult. Sci..

[18] Zhang W.-J., Xu Z.-R., Zhao S.-H., Sun J.-Y., Yang X. (2007). Development of a microbial fermentation process for detoxification of gossypol in cottonseed meal. Anim. Feed Sci. Technol..

[19] Shi C., Zhang Y., Yin Y., Wang C., Lu Z., Wang F., Feng J., Wang Y. (2017). Amino acid and phosphorus digestibility of fermented corn-soybean meal mixed feed with *Bacillus subtilis* and *Enterococcus faecium* fed to pigs. J. Anim. Sci..

[20] AOAC (2006). Official Methods of Analysis.

[21] Buddrick O., Jones O.A.H., Cornell H.J., Small D.M. (2014). The influence of fermentation processes and cereal grains in wholegrain bread on reducing phytate content. J. Cereal. Sci..

[22] Hong K.J., Lee C.H., Kim S.W. (2004). *Aspergillus oryzae* GB-107 fermentation improves nutritional quality of food soybeans and feed soybean meals. J. Med. Food.

[23] Anderson J.M., Van Itallie C.M., Fanning A.S. (2004). Setting up a selective barrier at the apical junction complex. Curr. Opin. Cell Biol..

[24] Peterson L.W., Artis D. (2014). Intestinal epithelial cells: Regulators of barrier function and immune homeostasis. Nat. Rev. Immunol..

[25] Gu C., Pan H., Sun Z., Qin G. (2010). Effect of soybean variety on anti-nutritional factors content, and growth performance and nutrients metabolism in rat. Int. J. Mol. Sci..

[26] Chi C.-H., Cho S.-J. (2016). Improvement of bioactivity of soybean meal by solid-state fermentation with *Bacillus amyloliquefaciens* versus *Lactobacillus* spp. and *Saccharomyces cerevisiae*. LWT Food Sci. Technol..

[27] Varsha K.K., Priya S., Devendra L., Nampoothiri K.M. (2014). Control of spoilage fungi by protective lactic acid bacteria displaying probiotic properties. Appl. Biochem. Biotechnol..

[28] Shi C., Zhang Y., Lu Z., Wang Y. (2017). Solid-state fermentation of corn-soybean meal mixed feed with *Bacillus subtilis* and *Enterococcus faecium* for degrading antinutritional factors and enhancing nutritional value. J. Anim. Sci. Biotechnol..

[29] Sokrab A.M., Mohamed Ahmed I.A., Babiker E.E. (2014). Effect of fermentation on antinutrients, and total and extractable minerals of high and low phytate corn genotypes. J. Food Sci. Technol..

[30] Livak K.J., Schmittgen T.D. (2001). Analysis of relative gene expression data using real-time quantitative PCR and the 2(-Delta Delta C(T)) Method. Methods.

[31] Hu J., Lu W., Wang C., Zhu R., Qiao J. (2008). Characteristics of Solid-state Fermented Feed and its Effects on Performance and Nutrient Digestibility in Growing-finishing Pigs. Asian Australs. J. Anim. Sci..

[32] Silk D.B., Chung Y.C., Berger K.L., Conley K., Beigler M., Sleisenger M.H., Spiller G.A., Kim Y.S. (1979). Comparison of oral feeding of peptide and amino acid meals to normal human subjects. Gut.

[33] Missotten J.A., Michiels J., Dierick N., Ovyn A., Akbarian A., De Smet S. (2013). Effect of fermented moist feed on performance, gut bacteria and gut histo-morphology in broilers. Br. Poult. Sci..

[34] Li L., Li W.F., Liu S.Z., Wang H.H. (2020). Probiotic fermented feed improved the production, health and nutrient utilisation of yellow-feathered broilers reared in high altitude in Tibet. Br. Poult. Sci..

[35] Caspary W.F. (1992). Physiology and pathophysiology of intestinal absorption. Am. J. Clin. Nutr..

[36] Xu Z.R., Hu C.H., Xia M.S., Zhan X.A., Wang M.Q. (2003). Effects of dietary fructooligosaccharide on digestive enzyme activities, intestinal microflora and morphology of male broilers. Poult. Sci..

[37] Jazi V., Foroozandeh A.D., Toghyani M., Dastar B., Rezaie Koochaksaraie R., Toghyani M. (2018). Effects of *Pediococcus acidilactici*, mannan-oligosaccharide, butyric acid and their combination on growth performance and intestinal health in young broiler chickens challenged with Salmonella Typhimurium. Poult. Sci..

[38] Zarkadas L.N., Wiseman J. (2005). Influence of processing of full fat soya beans included in diets for piglets. Anim. Feed. Sci. Technol..

[39] Kumar N., Arthur C.P., Ciferri C., Matsumoto M.L. (2020). Structure of the secretory immunoglobulin A core. Science.

[40] Mitjans M., Barniol G., Ferrer R. (1997). Mucosal surface area in chicken small intestine during development. Cell Tissue Res..

[41] Schneeberger E.E., Lynch R.D. (2004). The tight junction: A multifunctional complex. Am. J. Physiol. Cell Physiol..

[42] Awad W.A., Hess C., Hess M. (2017). Enteric Pathogens and Their Toxin-Induced Disruption of the Intestinal Barrier through Alteration of Tight Junctions in Chickens. Toxins.

[43] Johansson M.E., Larsson J.M., Hansson G.C. (2011). The two mucus layers of colon are organized by the MUC2 mucin, whereas the outer layer is a legislator of host-microbial interactions. Proc. Natl. Acad. Sci. USA.

[44] Gharib-Naseri K., de Paula Dorigam J.C., Doranalli K., Kheravii S., Swick R.A., Choct M., Wu S.B. (2020). Modulations of genes related to gut integrity, apoptosis, and immunity underlie the beneficial effects of *Bacillus amyloliquefaciens* CECT 5940 in broilers fed diets with different protein levels in a necrotic enteritis challenge model. J. Anim. Sci. Biotechnol..

[45] Pham V.H., Kan L., Huang J., Geng Y., Zhen W., Guo Y., Abbas W., Wang Z. (2020). Dietary encapsulated essential oils and organic acids mixture improves gut health in broiler chickens challenged with necrotic enteritis. J. Anim. Sci. Biotechnol..

[46] Ju T., Kong J.Y., Stothard P., Willing B.P. (2019). Defining the role of Parasutterella, a previously uncharacterized member of the core gut microbiota. ISME J..

[47] Eeckhaut V., Wang J., Van Parys A., Haesebrouck F., Joossens M., Falony G., Raes J., Ducatelle R., Van Immerseel F. (2016). The Probiotic *Butyricicoccus pullicaecorum* Reduces Feed Conversion and Protects from Potentially Harmful Intestinal Microorganisms and Necrotic Enteritis in Broilers. Front. Microbiol..

[48] Stanley D., Denman S.E., Hughes R.J., Geier M.S., Crowley T.M., Chen H., Haring V.R., Moore R.J. (2012). Intestinal microbiota associated with differential feed conversion efficiency in chickens. Appl. Microbiol. Biotechnol..

[49] Neubauer C., De Souza-Pilz M., Bojesen A.M., Bisgaard M., Hess M. (2009). Tissue distribution of haemolytic *Gallibacterium anatis* isolates in laying birds with reproductive disorders. Avian Pathol..

[50] Downes J., Dewhirst F.E., Tanner A.C.R., Wade W.G. (2013). Description of *Alloprevotella rava* gen. nov., sp. nov., isolated from the human oral cavity, and reclassification of *Prevotella tannerae* Moore et al. 1994 as *Alloprevotella tannerae* gen. nov., comb. nov.. Int. J. Syst. Evol. Microbiol..

[51] Feng J., Lu M., Wang J., Zhang H., Qiu K., Qi G., Wu S. (2021). Dietary oregano essential oil supplementation improves intestinal functions and alters gut microbiota in late-phase laying hens. J. Anim. Sci. Biotechnol..

[52] Wiersema M.L., Koester L.R., Schmitz-Esser S., Koltes D.A. (2021). Comparison of intestinal permeability, morphology, and ileal microbial communities of commercial hens housed in conventional cages and cage-free housing systems. Poult. Sci..

